# Supplementary figures and images for: Tim-3 is dispensable for allergic inflammation and respiratory tolerance in experimental asthma
Source: PLoS One. 2021 Apr 6;16(4):e0249605. doi: 10.1371/journal.pone.0249605 (PMC8023500; doi:10.1371/journal.pone.0249605)

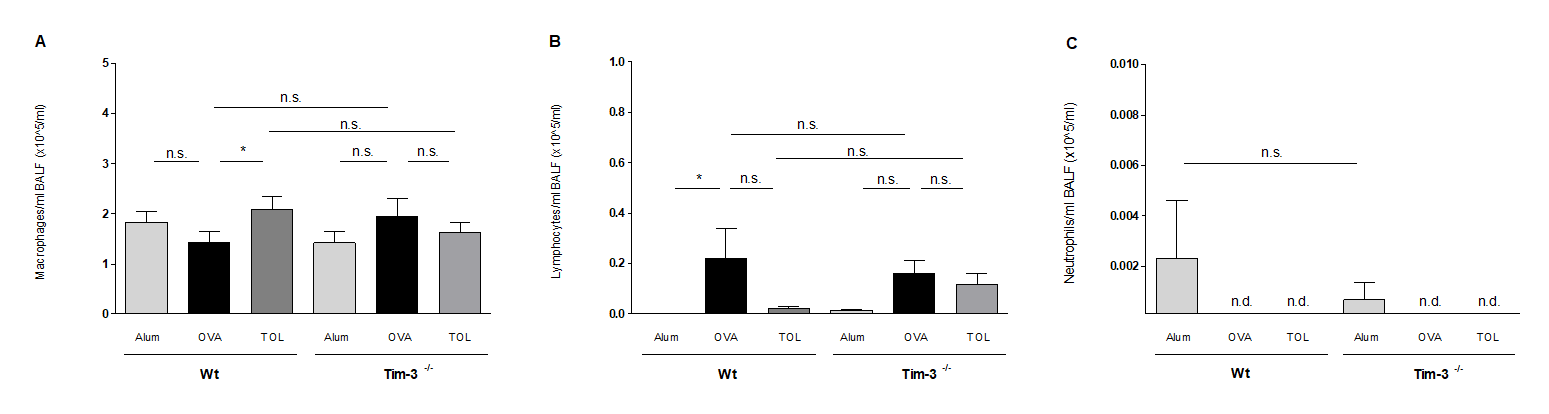

Supplement: S1 Fig — (A-C) BALF differential cell count was determined as number of macrophages (A), lymphocytes (B) and neutrophils (C) on cytospins. No significant differences were detected between WT and Tim-3-/- OVA and TOL mice, resulting in comparable allergic and tolerant phenotypes. (Data of three independent experiments; n = 12-15 animals per group.) Mann-Whitney-U-test. * p ≤ 0.05; ** p ≤ 0.01. Data are presented as mean ± SEM. (TIF) [file pone.0249605.s001.tif]

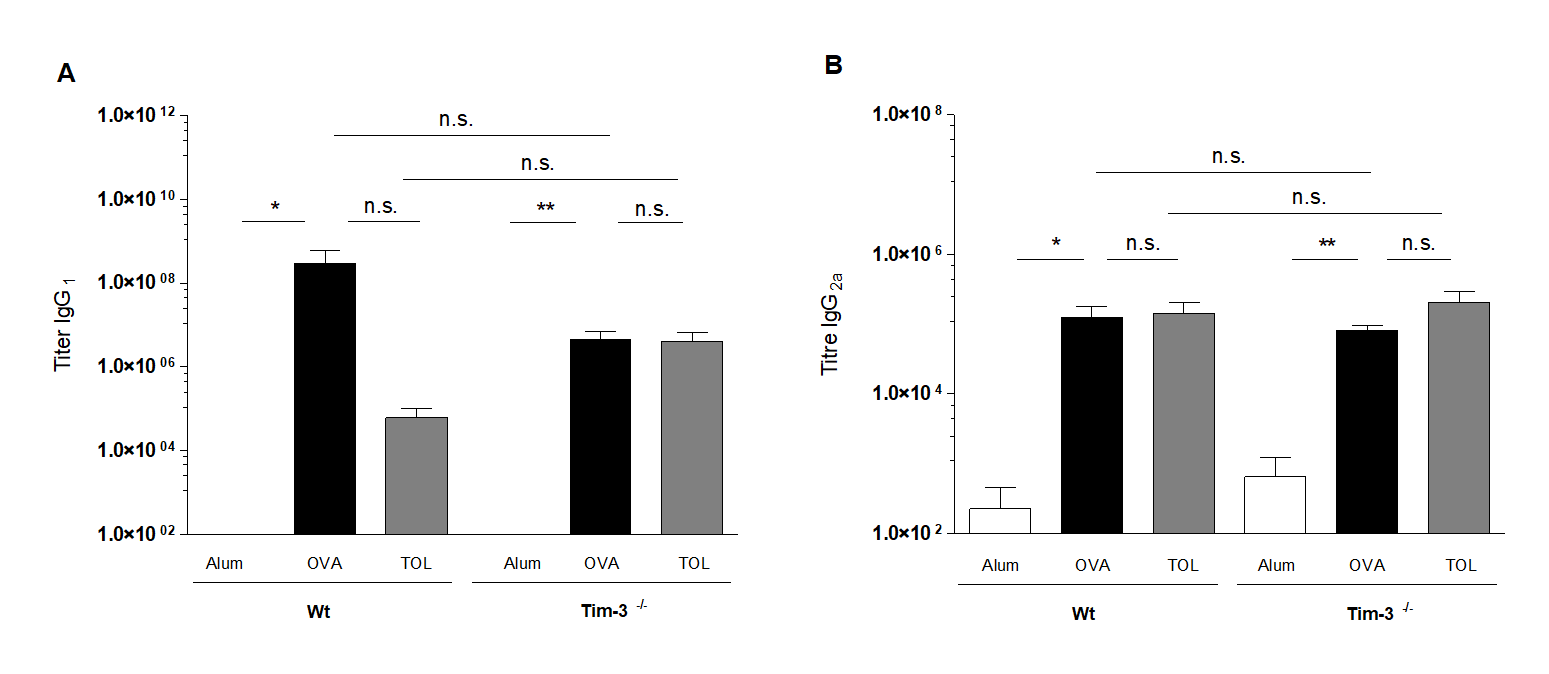

Supplement: S2 Fig — (A, B) OVA-specific IgG1 (A) and IgG2a (B) serum levels in WT and Tim-3-/- mice were not significantly altered in allergic vs. tolerant animals. No difference in WT vs. Tim-3-/- mice. (One representative out of three independent experiments; n = 5-6 animals for each group.) Mann-Whitney-U-test. * p ≤ 0.05; ** p ≤ 0.01. Data are presented as mean ± SEM. (TIF) [file pone.0249605.s002.tif]

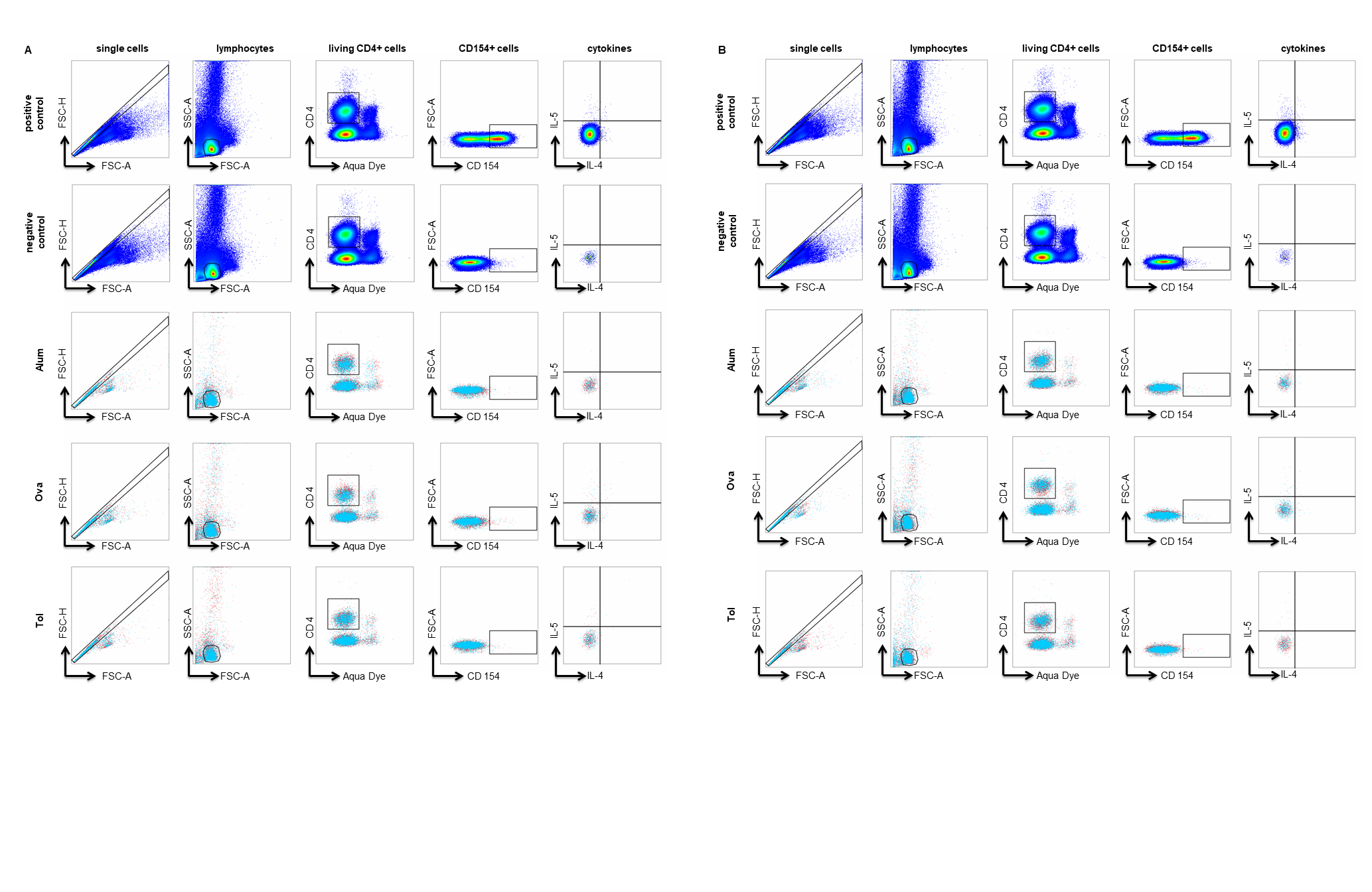

Supplement: S3 Fig — (A, B) Progressive gating strategy for identification of functional T cell subsets. After in vitro restimulation, splenocytes from WT (A) and Tim-3-/- (B) mice were incubated with amine-reactive viability dye to identify dead cells, followed by staining for CD4, fixation and intracellular staining for CD154, IFNγ, IL-17A, IL-4 and IL-5. Cells were gated through a singlet cell gate, followed by a lymphocyte gate and dead cell exclusion. T helper cells were identified using CD4 and OVA-specific T-cells were then gated using CD154 expression. The latter were then analyzed for cytokine production showing IL-4 and IL-5 here. (TIF) [file pone.0249605.s003.tif]

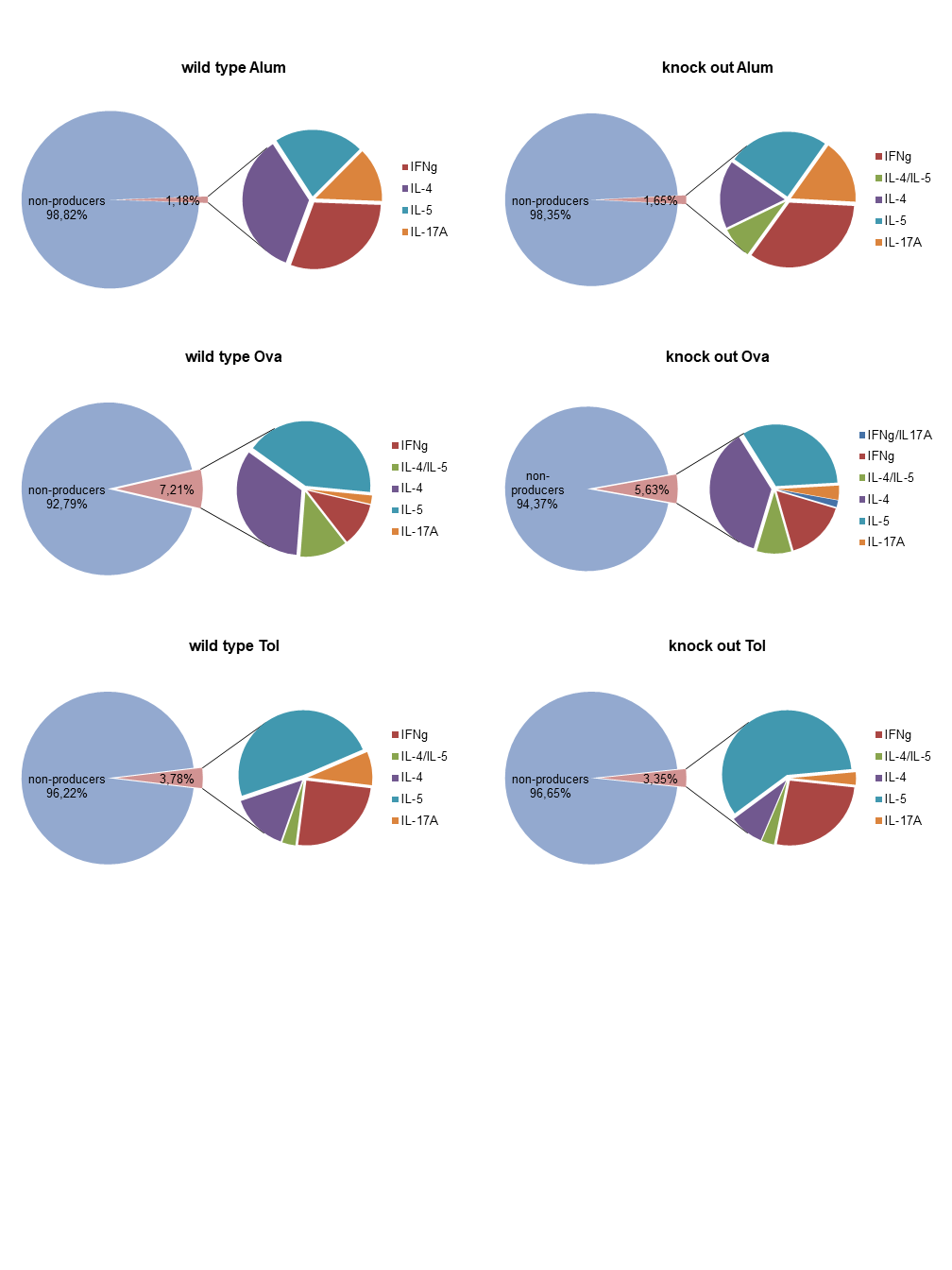

Supplement: S4 Fig — CD4+ cells were examined flow cytometrically for their production of IFNg, IL-4, IL-5, and IL-17A. The larger pie charts (light blue) show the percentage of CD4+ cells that produce any of the cytokines investigated. The smaller pie charts summarize the fractions of the total response that are single-producers of any of the individual cytokines or double producers that produce two cytokines simultaneously (e.g. IL-4/IL-5 or IFNg/IL-17). For example 7,21% of the CD4+ cells from WT OVA mice produced at least one of the investigated cytokines upon recognizing OVA. Of those, the majority was single producers of either IL-4 or IL-5 and only a small fraction produced IL-17. Responses are grouped and color-coded according to the number of functions. (TIF) [file pone.0249605.s004.tif]

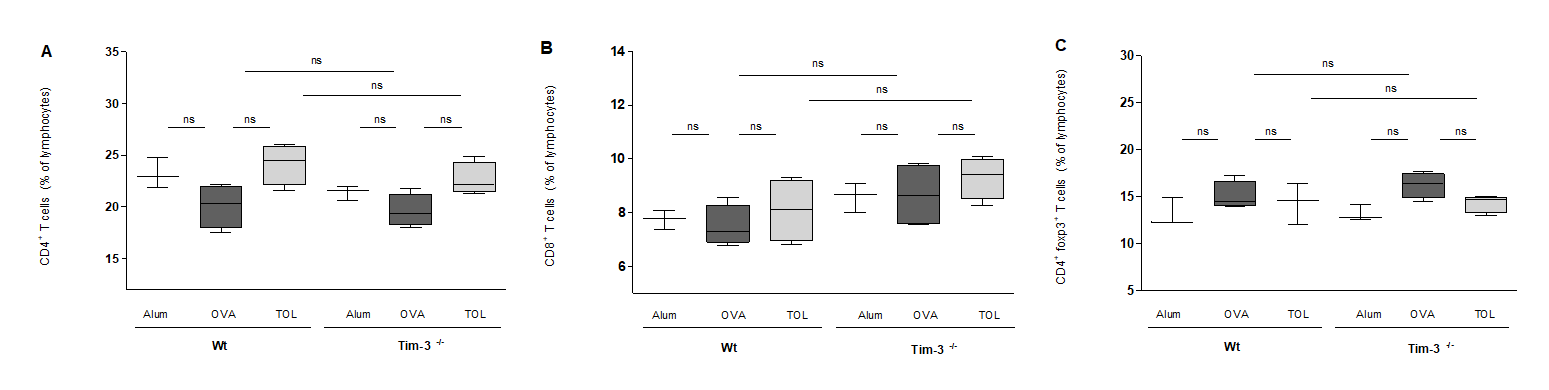

Supplement: S5 Fig — (A, B) Splenic single-cell suspensions were first flow cytometrically examined for their percentage of CD4+ (A) and CD8+ T cells (B). (C) After, intracellular staining of Foxp3 was performed. No statistically significant difference was detected between the percentage of different T cell subsets in OVA immunized and challenged mice compared to their non-allergic controls. In addition, Tim-3-/- mice did not differ in their composition of CD8+, CD4+ or CD4+Foxp3+ T cells. Mann-Whitney-U-test. ns p > 0.05. (TIF) [file pone.0249605.s005.tif]
