# Supplementary material for: Tim-3 is dispensable for allergic inflammation and respiratory tolerance in experimental asthma
Source: PLoS One. 2021 Apr 6;16(4):e0249605. doi: 10.1371/journal.pone.0249605 (PMC8023500; doi:10.1371/journal.pone.0249605)
Supplement: Attachment — Submitted filename: Response to Reviewers.docx [file pone.0249605.s006.docx]

1. **Response to Journal**

**Journal Requirements:**

When submitting your revision, we need you to address these additional requirements.

1.) Please ensure that your manuscript meets PLOS ONE's style requirements, including those for file naming.

File and figure naming was changed accordingly to meet PLOS ONE`s style requirements throughout the manuscript.

2.) In your Methods section, please provide additional information on the animal research and ensure you have included details on: (i) methods of sacrifice (ii) methods of anesthesia and/or analgesia, and (iii) efforts to alleviate suffering.

We provided additional information on the animal research and included (i) methods of sacrifice (ii) methods of anesthesia and/or analgesia, and (iii) efforts to alleviate suffering *(page 5, lines 101-105)*. In addition, we complemented supporting information and literature on the genetic background of Tim-3^-/-^ mice *(page 5, lines 94-98)*.

3.) Please upload a new copy of Figures S1 and S5 as the detail is not clear.

We uploaded new copies of Figures S1 and S5. All figures were approved by PACE tool. Please let us know if there are still any other concerns about the quality of the figures.

2. **Response to Editor**

**Additional Editor Comments:**

figures should be of publication quality, please revise and submit new files.

We uploaded new copies of all figures. All figures were approved by PACE tool. Please let us know if there are still any other concerns about the quality of the figures.

3. **Response to Reviewer**

**Reviewers' comments:**

Reviewer's Responses to Questions

**Comments to the Author**

Reviewer #1: This is a rather straightforward study that examines the impact of Tim-3 deficiency in mice on models of allergic asthma. It appears that the mouse line used is a germ line Tim-3 KO, so thus the work addresses the overall effects of total Tim-3 deficiency on the particular in vivo models. The overall finding here is that Tim-3 is not required for development of allergic airway inflammation, at least in response to the model antigen OVA, plus alum. Use of the the model antigen, rather than an actual allergen is a caveat here. In any case, the authors also use a tolerance-promoting regimen and again show that Tim-3 is not required for induction of tolerance to OVA in the airways. These analyses, in both the inflammatory and tolerance models, were also extended to examine the production of Th1 and Th2 cytokine. Although the results here are largely negative, this study is a nice addition to the literature and appropriate for this journal. There are a few things that I would like the authors to address:

1. It is not at all clear from the manuscript how the Tim-3 KO mice were generated. Which background? Which exons(s) knocked out?

We added a thorough description of the generation of Tim-3^-/-^ mice including supplemental literature (page 5, lines 94-98, (1)). In addition, Dr. Sebastian Drube who was responsible for the generation and breeding of mice was included in the list of authors.

2. The authors should comment on the production of IL-10 by Treg vs. Tr1 cells; which is the more important cell type in this model? On a related note, do Tr1 cells express Tim-3?

IL-10 is a cytokine secreted by a wide variety of cell types including DCs, mast cells, Th2 as well as Treg cells, and as pointed out by the reviewer specific “Tr1” cells. The expression of IL-10 is highly dynamic and tightly regulated. IL-10 exerts pleiotropic stimulatory and suppressive functionalities depending on the type of immune response analyzed *in vitro* and in *in vivo* model systems. To date, IL-10 is considered to have dual functions, not only as a prototypical anti-inflammatory cytokine but also as a stimulatory cytokine promoting immune responses, e.g. by supporting B-cell and CD8^+^ T-cell activation (2). As mentioned by the reviewer IL-10 is also secreted by “**Tr1**”cells (T regulatory type 1 cells, CD4+ foxp3-) that have been reported in the literature to be induced IL-10 dependently via DC-10 cells (3). Experimental studies by Matsuda and Nabe et al. have proven, that IL-10 secreting Foxp3- Tr1 cells generated *in vitro* or induced *in vivo* by SCIT (subcutaneous immunotherapy) are able to suppress allergic inflammation (4-6). So far, the *in vivo* expression pattern of these cells is poorly defined, besides biomarkers like CD49b or LAG-3 (7). To date, only limited data have been published on the expression of the inhibitory receptor Tim-3. White and Wraith et al. reported that human CD4(+)IL-10(+) T cells isolated *ex vivo*, following a short stimulation and cytokine secretion assay, contained significantly higher proportions of TIM-3(+) and PD-1(+) cells (7).

Up to date, we ourselves did not examine the Tr1 cell type in our OVA based allergic asthma mouse model, thus we can only generally comment on the reviewers question regarding IL-10 cytokine analysis:

We and others have shown that IL-10 is measurable in asthma or allergic disease mouse models (8,9), and observed that elevated IL-10 levels are associated with pronounced Th2 cell immune response after OVA immunization. On the other hand, experimental studies also reported that IL-10 can act suppressively as kind of a regulatory cytokine and controls Th2 cytokine secretion and eosinophilic inflammation. Furthermore, it is able to inhibit the synthesis of Th1 associated or pro-inflammatory cytokines. To date, it is known, that both Th1 and Th2 effector cells are capable to produce IL-10 after activation, this might be partially explained as a strategy of immune cells to limit their own activity and protect from damage caused by exaggerated inflammation (2,9-11).

Our present study did not focus on elucidating the exact role of the demonstrated elevated IL-10 levels after OVA immunization. Therefore, the increase of IL-10 might by a sign of effective Th2 cell immune stimulation or even in part sign of a counterbalancing cell activity. We did not clarify this further, based on the overall similar immune response of Tim3^-/-^ and WT mice. We observed decreased IL-10 levels in cell culture of splenocytes of tolerized mice (*TOL* group, Fig 3B) for both Tim-3^-/-^ and WT. In our setting, respiratory tolerance is rather mediated via Foxp3+ Treg cells, as demonstrated in a previous paper of our group (12). Busse et al. could show that CD4^+^CD25^+^Foxp3+ Treg numbers were increased in both spleen and lung after our i.n. OVA tolerization protocol and that Foxp3+ Treg cells isolated from *TOL* mice cultured in the presence of Dynabeads Mouse CD3/CD28 T Cell Expander^®^ can secrete IL-10. Busse et al. demonstrated by adoptive cell transfer experiment that these isolated Tregs conferred allergic asthma protection in our model.

We now added supporting information on the mechanism of tolerance induction in our model to highlight the role of Treg cells involved here (page 14-15, lines 322-328).

We apologize that we are not able to integrate new Tr1 or IL-10 related measurements into the present paper to clarify the reviewer question. However, the role of Tr1 cells might be of interest for future projects and we thank the reviewer for this valuable comment.

3. As stated above, OVA is not a natural allergen, so the authors should probably soften the global conclusion which suggests that Tim-3 is not required for allergic airway inflammation writ large.

To further emphasize limitations given by the usage of the allergen ovalbumin, we rephrased our conclusion on the significance of Tim-3 for *an OVA-based model* of allergic airway disease. We changed *Abstract* and *Discussion* sections correspondingly (*Abstract*, page 2, line 42; *Discussion,* page 18, line 412).

4. The figures are difficult to read, even the downloadable TIFF files, so I would like to see the figures at the proper resolution

Thank you very much for this valuable comment. We uploaded new figure files with a better resolution. All figures were approved by PACE tool. Please let us know if there are still any other concerns about the quality of the figures.

**References**

(1) Domenig C, Coyle AJ, Gutiérrez-Ramos J, Manlongat N, Kuchroo VK, Sánchez-Fueyo A, et al. Tim-3 inhibits T helper type 1-mediated auto- and alloimmune responses and promotes immunological tolerance. Nature Immunology 2003 Nov;4(11):1093-1101.

(2) Bedke T, Muscate F, Soukou S, Gagliani N, Huber S. IL-10-producing T cells and their dual functions. Seminars in immunology 2019 Aug;44:101335.

(3) Gregori S, Tomasoni D, Pacciani V, Scirpoli M, Battaglia M, Magnani CF, et al. Differentiation of type 1 T regulatory cells (Tr1) by tolerogenic DC-10 requires the IL-10–dependent ILT4/HLA-G pathway. Blood 2010 Aug 12,;116(6):935-944.

(4) Matsuda M, Doi K, Tsutsumi T, Inaba M, Hamaguchi J, Terada T, et al. Adoptive transfer of type 1 regulatory T cells suppressed the development of airway hyperresponsiveness in ovalbumin-induced airway inflammation model mice. Journal of pharmacological sciences 2019 Dec;141(4):139-145.

(5) Matsuda M, Morie Y, Oze H, Doi K, Tsutsumi T, Hamaguchi J, et al. Phenotype analyses of IL-10-producing Foxp3.sup.- CD4.sup.+ T cells increased by subcutaneous immunotherapy in allergic airway inflammation. International immunopharmacology 2018 Aug 1,;61:297.

(6) Matsuda M, Doi K, Tsutsumi T, Fujii S, Kishima M, Nishimura K, et al. Regulation of allergic airway inflammation by adoptive transfer of CD4+ T cells preferentially producing IL-10. European journal of pharmacology 2017 Oct 5,;812:38-47.

(7) White AM, Wraith DC. Tr1-like T cells – an enigmatic regulatory T cell lineage. Frontiers in immunology 2016;7:355.

(8) Polte T, Fuchs L, Behrendt A, Hansen G. Different role of CD30 in the development of acute and chronic airway inflammation in a murine asthma model. European journal of immunology 2009 Jul;39(7):1736-1742.

(9) Laouini D, Alenius H, Bryce P, Oettgen H, Tsitsikov E, Geha RS. IL-10 is critical for Th2 responses in a murine model of allergic dermatitis. The Journal of clinical investigation 2003 Oct 1,;112(7):1058-1066.

(10) Kosaka S, Tamauchi H, Terashima M, Maruyama H, Habu S, Kitasato H. IL-10 controls Th2-type cytokine production and eosinophil infiltration in a mouse model of allergic airway inflammation. Immunobiology (1979) 2011;216(7):811-820.

(11) Schülke S. Induction of Interleukin-10 Producing Dendritic Cells As a Tool to Suppress Allergen-Specific T Helper 2 Responses. Frontiers in immunology 2018;9:455.

(12) Busse M, Krech M, Meyer-Bahlburg A, Hennig C, Hansen G. ICOS Mediates the Generation and Function of CD4+CD25+Foxp3+ Regulatory T Cells Conveying Respiratory Tolerance. Journal of immunology (Baltimore, Md. : 1950) 2012 Aug 15,;189(4):1975-1982.
